# Supplementary material for: Resolving the full spectrum of human genome variation using Linked-Reads
Source: Genome Res. 2019 Apr;29(4):635–45. doi: 10.1101/gr.234443.118 (PMC6442396; doi:10.1101/gr.234443.118)
Supplement: Supplemental Material [file supp_gr.234443.118_Supplemental_longranger-2.2.zip › longranger-2.2.tar.bz2!/lr2.2-public/lib/stan/cmdstan/stan/doc/api/html/lkj__corr_8hpp_source.html]

Stan: src/stan/prob/distributions/multivariate/continuous/lkj\_corr.hpp Source File


|  |  |
| --- | --- |
|  | Stan  2.5.0  probability, sampling & optimization |


- Main Page
- Related Pages
- Namespaces
- Classes
- Files

- File List
- File Members

All Classes Namespaces Files Functions Variables Typedefs Enumerations Enumerator Friends Macros Pages

- src
- stan
- prob
- distributions
- multivariate
- continuous

lkj\_corr.hpp

Go to the documentation of this file.

1 #ifndef STAN\_\_PROB\_\_DISTRIBUTIONS\_\_MULTIVARIATE\_\_CONTINUOUS\_\_LKJ\_CORR\_HPP

2 #define STAN\_\_PROB\_\_DISTRIBUTIONS\_\_MULTIVARIATE\_\_CONTINUOUS\_\_LKJ\_CORR\_HPP

3

4 #include <stan/prob/constants.hpp>

5 #include <stan/math/matrix\_error\_handling.hpp>

6 #include <stan/math/error\_handling.hpp>

7 #include <stan/prob/traits.hpp>

8 #include <stan/prob/distributions/univariate/continuous/beta.hpp>

9 #include <stan/prob/transform.hpp>

10

11 namespace stan {

12  namespace prob {

13

14  template <typename T\_shape>

15  T\_shape do\_lkj\_constant(const T\_shape& eta, const unsigned int& K) {

16

17  using stan::math::sum;

18  using stan::math::lgamma;

19

20  // Lewandowski, Kurowicka, and Joe (2009) theorem 5

21  T\_shape constant;

22  const int Km1 = K - 1;

23  if (eta == 1.0) {

24  // C++ integer division is appropriate in this block

25  Eigen::VectorXd numerator( Km1 / 2 );

26  for(size\_t k = 1; k <= numerator.rows(); k++)

27  numerator(k-1) = lgamma(2 \* k);

28  constant = sum(numerator);

29  if ( (K % 2) == 1 ) constant += 0.25 \* (K \* K - 1) \* LOG\_PI -

30  0.25 \* (Km1 \* Km1) \* LOG\_TWO - Km1 \* lgamma( (K + 1) / 2);

31  else constant += 0.25 \* K \* (K - 2) \* LOG\_PI +

32  0.25 \* (3 \* K \* K - 4 \* K) \* LOG\_TWO +

33  K \* lgamma(K / 2) - Km1 \* lgamma(K);

34  }

35  else {

36  constant = -Km1 \* lgamma(eta + 0.5 \* Km1);

37  for (size\_t k = 1; k <= Km1; k++)

38  constant += 0.5 \* k \* LOG\_PI + lgamma(eta + 0.5 \* (Km1 - k));

39  }

40  return constant;

41  }

42

43  // LKJ\_Corr(L|eta) [ L Cholesky factor of correlation matrix

44  // eta > 0; eta == 1 <-> uniform]

45  template <bool propto,

46  typename T\_covar, typename T\_shape>

47  typename boost::math::tools::promote\_args<T\_covar, T\_shape>::type

48  lkj\_corr\_cholesky\_log(

49  const Eigen::Matrix<T\_covar,Eigen::Dynamic,Eigen::Dynamic>& L,

50  const T\_shape& eta) {

51

52  static const char\* function

53  = "stan::prob::lkj\_corr\_cholesky\_log(%1%)";

54

55  using boost::math::tools::promote\_args;

56  using stan::math::check\_positive;

57  using stan::math::check\_lower\_triangular;

58  using stan::math::sum;

59

60  typename promote\_args<T\_covar,T\_shape>::type lp(0.0);

61  check\_positive(function, eta, "Shape parameter", &lp);

62  check\_lower\_triangular(function, L, "Random variable", &lp);

63

64  const unsigned int K = L.rows();

65  if (K == 0)

66  return 0.0;

67

68  if (include\_summand<propto,T\_shape>::value)

69  lp += do\_lkj\_constant(eta, K);

70  if (include\_summand<propto,T\_covar,T\_shape>::value) {

71  const int Km1 = K - 1;

72  Eigen::Matrix<T\_covar,Eigen::Dynamic,1> log\_diagonals =

73  L.diagonal().tail(Km1).array().log();

74  Eigen::Matrix<T\_covar,Eigen::Dynamic,1> values(Km1);

75  for (size\_t k = 0; k < Km1; k++)

76  values(k) = (Km1 - k - 1) \* log\_diagonals(k);

77  if ( (eta == 1.0) &&

78  stan::is\_constant<typename stan::scalar\_type<T\_shape> >::value) {

79  lp += sum(values);

80  return(lp);

81  }

82  values += (2.0 \* eta - 2.0) \* log\_diagonals;

83  lp += sum(values);

84  }

85

86  return lp;

87  }

88

89  template <typename T\_covar, typename T\_shape>

90  inline

91  typename boost::math::tools::promote\_args<T\_covar, T\_shape>::type

92  lkj\_corr\_cholesky\_log(

93  const Eigen::Matrix<T\_covar,Eigen::Dynamic,Eigen::Dynamic>& L,

94  const T\_shape& eta) {

95  return lkj\_corr\_cholesky\_log<false>(L,eta);

96  }

97

98  // LKJ\_Corr(y|eta) [ y correlation matrix (not covariance matrix)

99  // eta > 0; eta == 1 <-> uniform]

100  template <bool propto,

101  typename T\_y, typename T\_shape>

102  typename boost::math::tools::promote\_args<T\_y, T\_shape>::type

103  lkj\_corr\_log(const Eigen::Matrix<T\_y,Eigen::Dynamic,Eigen::Dynamic>& y,

104  const T\_shape& eta) {

105  static const char\* function = "stan::prob::lkj\_corr\_log(%1%)";

106

107  using stan::math::check\_size\_match;

108  using stan::math::check\_not\_nan;

109  using stan::math::check\_positive;

110  using stan::math::check\_corr\_matrix;

111  using stan::math::sum;

112  using boost::math::tools::promote\_args;

113

114  typename promote\_args<T\_y,T\_shape>::type lp(0.0);

115  check\_positive(function, eta, "Shape parameter", &lp);

116  check\_size\_match(function,

117  y.rows(), "Rows of correlation matrix",

118  y.cols(), "columns of correlation matrix",

119  &lp);

120  check\_not\_nan(function, y, "Correlation matrix", &lp);

121  check\_corr\_matrix(function, y, "Correlation matrix", &lp);

122

123  const unsigned int K = y.rows();

124  if (K == 0)

125  return 0.0;

126

127  if (include\_summand<propto,T\_shape>::value)

128  lp += do\_lkj\_constant(eta, K);

129

130  if ( (eta == 1.0) &&

131  stan::is\_constant<typename stan::scalar\_type<T\_shape> >::value )

132  return lp;

133

134  if (!include\_summand<propto,T\_y,T\_shape>::value)

135  return lp;

136

137  Eigen::Matrix<T\_y,Eigen::Dynamic,1> values =

138  y.ldlt().vectorD().array().log().matrix();

139  lp += (eta - 1.0) \* sum(values);

140  return lp;

141  }

142

143  template <typename T\_y, typename T\_shape>

144  inline

145  typename boost::math::tools::promote\_args<T\_y, T\_shape>::type

146  lkj\_corr\_log(const Eigen::Matrix<T\_y,Eigen::Dynamic,Eigen::Dynamic>& y,

147  const T\_shape& eta) {

148  return lkj\_corr\_log<false>(y,eta);

149  }

150

151  template <class RNG>

152  inline Eigen::MatrixXd

153  lkj\_corr\_cholesky\_rng(const size\_t K,

154  const double eta,

155  RNG& rng) {

156  static const char\* function

157  = "stan::prob::lkj\_corr\_cholesky\_rng(%1%)";

158

159  using stan::math::check\_positive;

160

161  check\_positive(function, eta, "Shape parameter", (double\*)0);

162

163  Eigen::ArrayXd CPCs( (K \* (K - 1)) / 2 );

164  double alpha = eta + 0.5 \* (K - 1);

165  unsigned int count = 0;

166  for (size\_t i = 0; i < (K - 1); i++) {

167  alpha -= 0.5;

168  for (size\_t j = i + 1; j < K; j++) {

169  CPCs(count) = 2.0 \* stan::prob::beta\_rng(alpha,alpha,rng) - 1.0;

170  count++;

171  }

172  }

173  return stan::prob::read\_corr\_L(CPCs, K);

174  }

175

176  template <class RNG>

177  inline Eigen::MatrixXd

178  lkj\_corr\_rng(const size\_t K,

179  const double eta,

180  RNG& rng) {

181

182  static const char\* function

183  = "stan::prob::lkj\_corr\_rng(%1%)";

184

185  using stan::math::check\_positive;

186

187  check\_positive(function, eta, "Shape parameter", (double\*)0);

188

189  using stan::math::multiply\_lower\_tri\_self\_transpose;

190  return multiply\_lower\_tri\_self\_transpose(

191  lkj\_corr\_cholesky\_rng(K, eta, rng) );

192  }

193

194  }

195 }

196 #endif

stan::prob::lkj\_corr\_cholesky\_rng

Eigen::MatrixXd lkj\_corr\_cholesky\_rng(const size\_t K, const double eta, RNG &rng)

**Definition:** lkj\_corr.hpp:153

stan::is\_constant

Metaprogramming struct to detect whether a given type is constant in the mathematical sense (not the ...

**Definition:** traits.hpp:43

stan::scalar\_type

Metaprogram structure to determine the base scalar type of a template argument.

**Definition:** traits.hpp:138

beta.hpp

traits.hpp

stan::prob::lkj\_corr\_cholesky\_log

boost::math::tools::promote\_args< T\_covar, T\_shape >::type lkj\_corr\_cholesky\_log(const Eigen::Matrix< T\_covar, Eigen::Dynamic, Eigen::Dynamic > &L, const T\_shape &eta)

**Definition:** lkj\_corr.hpp:48

stan::math::check\_size\_match

bool check\_size\_match(const char \*function, T\_size1 i, const char \*name\_i, T\_size2 j, const char \*name\_j, T\_result \*result)

**Definition:** check\_size\_match.hpp:13

error\_handling.hpp

stan::prob::do\_lkj\_constant

T\_shape do\_lkj\_constant(const T\_shape &eta, const unsigned int &K)

**Definition:** lkj\_corr.hpp:15

matrix\_error\_handling.hpp

stan::prob::lkj\_corr\_log

boost::math::tools::promote\_args< T\_y, T\_shape >::type lkj\_corr\_log(const Eigen::Matrix< T\_y, Eigen::Dynamic, Eigen::Dynamic > &y, const T\_shape &eta)

**Definition:** lkj\_corr.hpp:103

constants.hpp

stan::agrad::sum

fvar< T > sum(const Eigen::Matrix< fvar< T >, R, C > &m)

**Definition:** sum.hpp:14

stan::agrad::lgamma

fvar< T > lgamma(const fvar< T > &x)

**Definition:** lgamma.hpp:15

stan::prob::include\_summand

Template metaprogram to calculate whether a summand needs to be included in a proportional (log) prob...

**Definition:** traits.hpp:35

stan::prob::lkj\_corr\_rng

Eigen::MatrixXd lkj\_corr\_rng(const size\_t K, const double eta, RNG &rng)

**Definition:** lkj\_corr.hpp:178

stan::math::lgamma

double lgamma(double x)

**Definition:** lgamma.hpp:31

stan::prob::read\_corr\_L

Eigen::Matrix< T, Eigen::Dynamic, Eigen::Dynamic > read\_corr\_L(const Eigen::Array< T, Eigen::Dynamic, 1 > &CPCs, const size\_t K)

Return the Cholesky factor of the correlation matrix of the specified dimensionality corresponding to...

**Definition:** transform.hpp:139

stan::math::check\_positive

bool check\_positive(const char \*function, const T\_y &y, const char \*name, T\_result \*result)

**Definition:** check\_positive.hpp:54

stan::math::check\_corr\_matrix

bool check\_corr\_matrix(const char \*function, const Eigen::Matrix< T\_y, Eigen::Dynamic, Eigen::Dynamic > &y, const char \*name, T\_result \*result)

Return true if the specified matrix is a valid correlation matrix.

**Definition:** check\_corr\_matrix.hpp:38

stan::prob::beta\_rng

double beta\_rng(const double alpha, const double beta, RNG &rng)

**Definition:** beta.hpp:593

stan::math::sum

double sum(std::vector< double > &x)

**Definition:** sum.hpp:10

transform.hpp

stan::math::check\_not\_nan

bool check\_not\_nan(const char \*function, const T\_y &y, const char \*name, T\_result \*result)

Checks if the variable y is nan.

**Definition:** check\_not\_nan.hpp:56

stan::agrad::multiply\_lower\_tri\_self\_transpose

Eigen::Matrix< fvar< T >, R, R > multiply\_lower\_tri\_self\_transpose(const Eigen::Matrix< fvar< T >, R, C > &m)

**Definition:** multiply\_lower\_tri\_self\_transpose.hpp:17

stan::math::multiply\_lower\_tri\_self\_transpose

matrix\_d multiply\_lower\_tri\_self\_transpose(const matrix\_d &L)

Returns the result of multiplying the lower triangular portion of the input matrix by its own transpo...

**Definition:** multiply\_lower\_tri\_self\_transpose.hpp:18

stan::math::check\_lower\_triangular

bool check\_lower\_triangular(const char \*function, const Eigen::Matrix< T\_y, Eigen::Dynamic, Eigen::Dynamic > &y, const char \*name, T\_result \*result)

Return true if the specified matrix is lower triangular.

**Definition:** check\_lower\_triangular.hpp:27


---

[ Stan Home Page ]

*© 2011–2014,
Stan Development Team.*
